# Salivary Cortisol as a Biomarker to Explore the Role of Maternal Stress in Early Childhood Caries

**DOI:** 10.1155/2013/565102

**Published:** 2013-05-28

**Authors:** Sharat Chandra Pani, Deena Abuthuraya, Hadia M. AlShammery, Dalal AlShammery, Hind AlShehri

**Affiliations:** ^1^Division of Pediatric and Preventive Dentistry, Riyadh Colleges of Dentistry and Pharmacy, P.O. Box 84891, Riyadh 11681, Saudi Arabia; ^2^Riyadh Colleges of Dentistry and Pharmacy, P.O. Box 84891, Riyadh 11681, Saudi Arabia

## Abstract

*Objective*. To compare salivary cortisol levels of children with ECC and their mothers with those of caries free children from a similar sociodemographic cohort. *Design*. Sixty-four college-educated, working mothers from middle income families with no history of anxiety disorders and their first born children aged between 48 and 71 months were included in the study. Salivary cortisol levels were analyzed using electrochemiluminescence (ECL) immunoassay. *Statistical Analyses*. Significance of difference between the cortisol levels of children with ECC and control children and of their mothers was analyzed using the Student's *t*- test. The intraclass correlation coefficient was used to measure the significance of correlation of cortisol levels between the mother and the child with logistic regression to explore possible associations. *Results*. Mothers of children with ECC had significantly higher levels of salivary cortisol (*P* < 0.05) than mothers of caries free children. The salivary cortisol levels of children with ECC were significantly higher than caries free children (*P* < 0.0001). A significant correlation existed between the salivary cortisol level of the mother and that of the child (*P* < 0.0001). *Conclusion*. While salivary cortisol levels of the child seem to have a direct impact on the incidence of ECC, maternal stress seems to have an indirect effect.

## 1. Introduction

Early childhood caries (ECC) has been defined as the occurrence of one or more carious lesions occurring in a child younger than 71 months of age [1]. Caries is a multifactorial disease, and among the many factors associated with ECC, the role of stress has recently received a lot of attention [2–5]. The relationship between caries and stress is not one of simple cause and effect and must be viewed in the context of socioeconomic factors [2]. 

Recently a unifying conceptual model has suggested that in addition to environmental and child factors maternal factors, such as stress in the mother, could contribute significantly to the development of early childhood caries [6]. While some authors have suggested a positive correlation between the stress of parents and caries in children [7, 8], others using the same methods have found no association between stress and caries [4]. The development of caries in children of mothers with high stress has been attributed to the poor parenting and feeding habits that are a result of stress [4, 6, 9]. It is known that stress in the parents, especially the mother, could be transmitted to their children [10]. However little attempt has been made to either quantify this stress using a biomarker or evaluate the correlation between the stress level of the parent and that of the child with relation to early childhood caries.

Salivary cortisol levels provide an accurate, reliable, and noninvasive measure of stress in both adults and children [11]. Cortisol is a hormone secreted by the hypothalamus pituitary adrenal axis (HPAA) and has been used as an accurate biomarker in stress research for over half a century [12]. In dentistry salivary cortisol has been used to measure the role of stress in a variety of settings from the anxiety of dental treatment [13, 14] to periodontal disease and dental caries [15, 16]. Children with ECC have constantly shown higher levels of salivary cortisol than their caries free counterparts suggesting that stress could influence the development of caries in children [2, 5]. 

The aim of this study was to study the relationship between maternal stress, childhood stress, and early childhood caries, among working mothers from middle income families and their first born children using salivary cortisol as a biomarker.

## 2. Methodology

Ethical clearance for the study was obtained from the research center of the Riyadh Colleges of Dentistry and Pharmacy (USRP-2011-010, December 23rd, 2011). Pamphlets in Arabic about the objectives of the study and the need for volunteers were circulated among working mothers in Riyadh city. A total of 106 mothers contacted the authors and were then screened to evaluate if they met the inclusion criteria. The procedure was explained to all selected mothers and they signed an informed consent form for themselves and on behalf of their child before participating in the study.

### 2.1. Selection of the Sample

Mothers were selected if they met the following inclusion criteria; they were from a middle income family (monthly household income between 14,000 and 20,000 SAR [US$ 3715–5305]); they had a college education and were working. The children were the first born children in the family were aged between 48 and 71 months of age and had one younger sibling. The children were attending preschool and were placed in day-care centers during the working hours of the mother. Mothers who had children with a history of systemic illness or long-term medication were excluded from the study. Mothers who were themselves on antipsychotic medication or had a history of psychiatric illness were excluded from the study. Single mothers were excluded from the study. A total of 64 mothers and their first born children met the inclusion criteria. 

### 2.2. Collection of Saliva

Saliva was collected using the passive drool method [17] into sterile collection tubes (Greiner Bio-One GmbH, Austria). These tubes were then stored in a vaccine box (Apex International, Uttar Pradesh, India) at 5°C before being transported to the lab for analysis.

Given the diurnal and intraweek variation in the levels of salivary cortisol [18], the samples were collected two hours after waking up on Wednesday mornings. To standardize the time of collection parents who agreed to participate in the study consented to wake up their children at 0700 hrs. A call was placed to the parents and children between 0700 and 0730 hrs on day of collection of saliva by one of the investigators to verify the time of waking up.

### 2.3. Analysis of Salivary Cortisol

Salivary cortisol was estimated using the electrochemiluminescence (ECL) immunoassay using an Elecsys e immunoassay analyzer and the Cobas Salivary Kit. (Roche Diagnostics GmbH, Mannheim, Germany).

### 2.4. Recording the Caries Experience

The dft of the children was recorded by one of the authors while another recorded the DMFT of the mothers. Examination was done in a dental chair with a mouth mirror and ball ended probe using WHO criteria for the diagnosis of a carious lesion. Bitewing radiographs of both the children and the mothers were taken to assess the presence of proximal caries.

### 2.5. Statistical Analyses

The power of the sample was calculated using the G-Power 3.1.3 power analysis software (Universtät Kiel, Germany). The minimum required sample for the intraclass correlation with an alpha of 0.05 and a one tailed model with an *r* of 0.3 was 110 (55 pairs). The achieved power of this sample of 64 pairs with alpha of 0.05 and a two tailed model for the paired *t* test was 0.976 with a critical *t* of 1.99.

The salivary cortisol data was subject to an analysis of skewness and the Shapiro-Wilk test for significance of skew. The cortisol data had a skewness of 1.83 and the Shapiro-Wilk statistic (0.893, *P* < 0.001) suggested a dataset with significant skew to the left.

Given the skew of cortisol values, nonparametric tests were used. The Mann-Whitney *u* test was used to measure the significance of difference in the cortisol level of children with and without ECC, as well as in the cortisol levels of mothers of children with ECC and mothers of children without ECC. A paired samples correlation, the intraclass correlation coefficient (ICC) was used to measure the association between the salivary cortisol levels of the children and that of their mothers. Spearman's correlation was used to explore the relationship between the salivary cortisol level and the severity of the caries. A logistic regression model was posited to explore the results of the correlation and examine the association between maternal cortisol levels, children's cortisol levels, and the presence of ECC.

All data was processed using the SPSS ver.20 data processing software.

## 3. Results 

The sample comprised 64 mothers aged between 24 and 31 years (mean age 27.2 ± 2.6 years). The children (34 boys and 30 girls) were aged between 49 months and 70 months (mean age 54.1 ± 8.2 months), 24 of whom were caries free, while 40 had ECC. The caries distribution of the children in the ECC group and the DMF of the mothers is tabulated in Table 1. The salivary cortisol levels of the mothers ranged between .093 *μ*g/dL and .995 *μ*g/dL (mean .358 ± .24 *μ*g/dL). The salivary cortisol levels of the children ranged from .012 *μ*g/dL to .54 *μ*g/dL (mean .165 ± .012 *μ*g/dL).

The mean salivary cortisol levels of children with ECC were significantly higher than caries free children. The mean salivary cortisol levels of mothers of children with ECC were also significantly higher than those of mothers of caries free children. (Table 2) No significant gender differences were found in the salivary cortisol levels of the children.

When the cortisol levels of the children were paired with those of their mothers, the intraclass correlation coefficient showed a highly significant correlation between the two. (Table 3) The Pearson's correlation showed significant correlation between salivary cortisol level of the child and the dft (*r* = 0.549, *P* < 0.0001) and between the salivary cortisol level of the mother and the DMFT of the mother (*r* = 0.349, *P* = 0.004). However there was no correlation between the salivary cortisol level of the mother and the dft of the child (*r* = 0.042, *P* = 0.840). As these correlations suggest an association between stress and the incidence of ECC rather than the severity of ECC, a binary logistic regression model was framed using the presence or absence of ECC as the dependent variable and the maternal and child salivary cortisol levels as covariates.

The regression model showed a likelihood ratio of 11.22 and a Nagelkerke *R* ^2^ of .814 suggesting excellent goodness of fit. The model suggested that significant associations existed between caries and the overall model, as well as caries and the salivary cortisol level of the child. Maternal cortisol levels, though positively associated with caries, did not show significant association in the model (Table 4).

## 4. Discussion

The association between stress, in both children and mothers, and ECC has been receiving considerable attention in recent literature [2, 6] with authors seeking to expand on existing “socio-dental” models [19–22]. Although the role of salivary cortisol, and thereby childhood stress, has been explored previously [2, 3, 5], there is little data on maternal stress and ECC. The goal of this study was to use salivary cortisol a biomarker to assess the impact of maternal stress on ECC in a selected socioeconomic group.

The role of social factors in dental caries in general and ECC in particular has been discussed in detail [2, 6, 19]. In order to detect the impact of stress on ECC we decided to choose a narrow social and economic construct for this study, reducing the effect of confounding factors. Poor education of the mother [23], low socioeconomic status [2, 20], being a single parent [24], and history of anxiety disorders [9, 10, 23] have all been shown to be risk factors for the development of caries and were thus made part of the exclusion criteria. Children of working mothers who were left at day-care centers were chosen for this study as it has been shown that the regulation of diet in the time the child is in the center actually helps to reduce the risk of caries [25].

The analysis of salivary cortisol is technique sensitive and highly sensitive to diurnal variation [11, 12]. In addition there is also evidence to show that the cortisol levels are their highest early in the morning and on the last day of the working week [18]. It was for these reasons that the collection of cortisol was scheduled one hour after waking up on Wednesday (last day of the working week in Saudi Arabia). The decision to use the passive drool method for collection of saliva was based on the work of Putnam et al. who showed this to be a simple and effective technique for the recovery of cortisol in children [17]. 

The finding of significantly higher cortisol levels in children with ECC is in keeping with those previous studies [2, 5] that indicate a higher cortisol levels in children with caries. The finding of significantly higher cortisol levels in the mothers of children with ECC is, however, a previously unreported finding. The role of maternal stress on the development of ECC has been previously reported and discussed [4, 6, 7, 9, 23]. The finding of higher cortisol levels in mothers of children with ECC lends support to the argument that maternal stress is an integral part of the pathogenesis of caries in children [4, 6, 7]. 

The increased level of caries in children of mothers with “high stress” has often been discussed in the context of mothers with stress disorders [20, 22], single mothers [24], or familial stress [9, 23]. However the exclusion of these “risk” groups from our study sample indicates that the role of stress may be more ubiquitous than previously anticipated. The strong correlation between the cortisol levels of the mother and those of the child is in keeping with recent anxiety research that maternal transmission of anxiety to their children may be a continuous process that has long-term implications [10]. 

Most psychosocial models of ECC assign an indirect role to maternal stress suggesting that the poor oral habits and lack of time for the child's oral health result in the development of caries [4, 9, 20, 22, 23]. The aim of the regression model used in this study was to identify the impact that cortisol levels of the child and the mother had on dental caries. The goodness of fit of the logistic regression model lends support to the assumption that maternal stress and the stress of the child are strongly linked to the presence of ECC in children. The significant association of dft with salivary cortisol in the child, coupled with a lack of significant association with maternal cortisol in the regression model, seems to support the theories that stress in the child could have a direct influence on the occurrence of ECC [2, 5] while also lending credence to the theory that maternal stress plays an indirect role in the development of caries [4, 6, 7, 23].

The results of this study however must be viewed within the limitations of the study itself. The use of a narrow social and economic construct in this study, while useful in demonstrating the role of maternal stress, means that the study would need to be expanded to view the impact across different socioeconomic strata. Furthermore the high prevalence of ECC in Saudi Arabia [26, 27] may actually mask the overall impact of maternal stress. Studies from countries with low caries rates could help explore in greater detail the impact of maternal stress.

## 5. Conclusion

Within the limitations of the narrow sociodemographic cohort of this study, we can conclude that a definite relationship exists between salivary cortisol levels of the mother, salivary cortisol levels of the child, and the development of ECC. A larger study that uses multiple sociodemographic groups and data from countries with varying prevalence of ECC could shed more light on the exact nature of the role of maternal stress.

## Figures and Tables

**Table 1:** Caries distribution of the study population.

	Group	Mean	Std. deviation	*t*	Sig
DMFT (mothers)	Control	12.6154	4.20549	−3.16	.002*
	ECC	15.7105	3.58607		

dft	Control	NA	NA	NA	NA
	ECC	5.8421	3.69460		

NA: Not applicable as the dft of the children in the control group was 0.

*Difference significant at *P* < 0.01, measured using the unpaired *t* test.

**Table 2:** Comparison of mean salivary cortisol levels in children and mothers of children with and without early childhood caries.

	Group	*N*	Mean	Std. deviation	*t*	Sig^a^
Salivary cortisol level-Mothers	ECC	40	.40645	.259784	2.144	.036*
	Caries free	24	.27904	.210454		

Salivary cortisol level-Children	ECC	40	.22403	.107102	7.925	.000**
	Caries free	24	.06742	.049905		

^a^Measured using the Mann-Whitney *U* test.

*Differences significant at *P* < 0.05.

**Differences significant at *P* < 0.001.

**Table 3:** Comparison of salivary cortisol levels of children when paired with their mothers.

	Intraclass correlation^a^	95% confidence interval		*F* Test with true value 0			
		Lower bound	Upper bound	Value	df1	df2	Sig
Single measures	.496	.286	.660	2.968	63	63	.000*
Average measures	.663	.445	.795	2.968	63	63	.000*

^a^Paired correlation between salivary cortisol level of the mother and the salivary cortisol level of the child tested using the intraclass correlation coefficient.

*Correlation significant at *P* < 0.001.

**Table:** (a)

Model summary			
	−2Log likelihood	Cox and Snell *R* ^2^	Nagelkerke *R* ^2^
	32.712	.556	.758

**Table:** (b)

Variables in the equation						
	*B*	S.E.	Wald	df	Sig.	Exp(*B*)
Cortisol-Mother	−3.146	2.216	2.015	1	.156	.043
Cortisol-Child	41.440	10.291	16.215	1	.000*	9.934
Constant	−3.853	1.192	10.448	1	.001*	.021

*Association significant at *P* < 0.001.
